# The expression of Methyltransferase-like 3 in dentigerous cyst and ameloblastoma

**DOI:** 10.4317/jced.62186

**Published:** 2024-12-01

**Authors:** Chatchaphan Udompatanakorn, Supisara Patcharaman, Patrayu Taebunpakul

**Affiliations:** 1 Department of Oral Surgery and Oral Medicine, Faculty of Dentistry, Srinakharinwirot University

## Abstract

**Background:**

N6-methyladenosine (m6A), the most abundant internal RNA modification in mammals, plays a critical role in many forms of cancer. Methyltransferase-like 3 (METTL3) serves as the main catalytic subunit of the m6A writer and plays a role in the progression of head and neck squamous cell carcinoma. To date, the role of METTL3 in odontogenic lesions has never been investigated. This study aimed to examine METTL3 expression in dental follicles (DF), dentigerous cysts (DC), unicystic ameloblastoma (UA), and conventional ameloblastoma (conventional AM).

**Material and Methods:**

The immunohistochemistry was performed using paraffin-embedded tissue samples. Seven cases of DF, 30 cases of DC, and 35 cases of AM (20 cases of UA and 15 cases of conventional AM) were included. The expression patterns, percentage of METTL3-positive cells, staining intensities, and immunoreactive scores (IRS) were examined.

**Results:**

The percentage of METTL3-positive cells was found to be significantly higher in the AM compared to DC and DF samples (*p*<0.01). Additionally, the percentage of METTL3-positive cells increased from the luminal/intraluminal subtype of UA, the mural subtype of UA, to the conventional AM (*p*<0.01). All AM samples had higher METTL3 cell staining intensity and IRS scores than the DF and DC samples (*p*<0.01). The mural subtype of UA and conventional AM also had significantly higher cell intensity and IRS scores than the luminal/intraluminal subtype of UA (*p*<0.05).

**Conclusions:**

The results of this study suggest that METTL3 might have a role in odontogenic lesions. METTL3 expression may be related to the aggressive behaviour of these lesions. However, the precise molecular mechanism of METTL3 in odontogenic lesions still needs to be elucidated.

** Key words:**Methyltransferase-like 3, immunohistochemistry, dentigerous cyst, ameloblastoma.

## Introduction

Odontogenic cysts and tumours are heterogeneous groups of lesions originating from the tissue remnants of the tooth-forming apparatus (1). A dentigerous cyst (DC) is one of the most common developmental odontogenic cysts involving embedded or impacted teeth (2). It originates from the accumulation of fluid between the tooth crown and reduced enamel epithelium (REE) (2). DC is usually asymptomatic, though it may cause bone destruction and transform into an odontogenic tumour (such as ameloblastoma (AM)) or a malignant odontogenic tumour (such as primary intraosseous squamous cell carcinoma) (2,3). AM is the most common benign odontogenic tumour of epithelial origin (4). It arises from multiple sources of odontogenic epithelium, such as dental lamina or REE (4). AM is a slow-growing, locally aggressive tumour, and up to 1% of AM may undergo malignant transformation (5). Currently, classification by the World Health Organisation (WHO) in 2022 categorises AM into the following five types: conventional AM, unicystic AM (UA), peripheral, adenoid, and metastasizing AM (6).

N6-methyladenosine (m6A) is the most common internal mRNA modification in mammals. m6A modification has been reported to regulate the metabolism of RNAs, such as RNA splicing, translation, and stability (7). Recently, m6A modification has been shown to be involved in various pathological conditions, especially in the progression of various forms of cancer (7). m6A modification is a reversible biological process and is controlled by 1) m6A writers, such as methyltransferase-like 3 (METTL3) and their co-factors, which can catalyse m6A modifications on RNA, 2) m6A erasers, which can remove the modifications, and 3) m6A readers, which can recognise m6A methylation (7,8). METTL3 serves as the main catalytic subunit of the methyltransferase complex and plays roles in cancer metabolism, either as an oncogene or a tumour suppressor gene (9). According to the Cancer Genome Atlas (TCGA) database, METTL3 expression is higher in head and neck squamous cell carcinoma (HNSCC) compared to normal tissue (10). Previous studies demonstrated that METTL3 expression was elevated in oral epithelial dysplasia and oral squamous cell carcinoma (OSCC) compared to normal oral mucosa (11,12). Moreover, METTL3 overexpression promotes OSCC cell proliferation, migration, and invasion *in vitro* (12,13).

The pathogenesis of odontogenic lesions remains largely unknown (14). Recently, Niu *et al*. (2020) suggested that m6A could be involved in the development of AM (8). To the best of the authors’ knowledge, no information exists regarding the roles of METTL3 in odontogenic lesions. This study aimed to investigate the expression of METTL3 expression in dental follicles (DF), dentigerous cysts (DC), unicystic ameloblastoma (UA), and conventional ameloblastoma (conventional AM) using immunohistochemistry. The findings may provide a better understanding of the roles of METTL3 in the pathogenesis of odontogenic lesions.

## Material and Methods

-Samples

A retrospective study was performed using a total of 72 formalin-fixed paraffin-embedded (FFPE) tissue samples retrieved from the Department of Oral Surgery and Oral Medicine, Faculty of Dentistry, Srinakharinwirot University, between December 2015 and December 2022. The specimens comprised seven cases of DF, 30 cases of DC, and 35 cases of AM (20 cases of UA and 15 cases of conventional AM). All samples were incisional biopsy specimens. The inclusion criteria were FFPE tissues of odontogenic lesions that had been histologically graded according to the 5th edition of the World Health Organisation Classification of Odontogenic Cysts and Tumours (15). The exclusion criteria were specimens without epithelial linings or nests, or specimens with intense inflammatory cells. An oral pathologist (CU) confirmed the histopathological diagnoses. The clinicopathologic characteristics of the lesions were collected from the chart records. This study was approved by the Ethical Committee for Human Research, Srinakharinwirot University (no. SWUEC-097/2566X).

-Immunohistochemistry

Briefly, the FFPE tissue samples were cut into 4 µM sections, deparaffinised, and rehydrated in a series of graded alcohols. The immunohistochemistry was performed using an EnVision kit (Dako Agilent, California, USA). Antigen retrieval was conducted using a microwave (700 W) with a Tris-EDTA buffer at pH 9.0 for 10 min. After cooling for 20 min to room temperature, endogenous peroxidase activity was blocked for 10 min. The sections were then washed in a wash buffer and incubated with 5% bovine serum albumin (BSA) for 10 min to block nonspecific activity. The sections were then incubated with anti-METTL3 primary antibody (ab195352, Abcam, Cambridge, UK) overnight at 4°C with a 1:250 dilution. After washing with a wash buffer, the sections were incubated with a secondary antibody for 30 min. The sections were then washed and stained with 3,3’-diaminobenzidine (DAB) for 3 min and subsequently washed in tap water, counterstained with Mayer’s hematoxylin, dehydrated, and mounted with coverslips. Human bladder cancer tissue was used as a positive control (16). The negative control was obtained by using 5% BSA without the primary antibody.

-Interpretation of METTL3 immunostaining

The METTL3 expression patterns, percentage of positive cells, cell staining intensities, and immunoreactive scores (IRS) (17) were recorded. In each slide, five random areas of epithelial components were photographed using EPview version 1.4 under a light microscope (Olympus, Tokyo, Japan). A total of at least 500 epithelial cells on each sample were counted. The photos were then analysed at 400x magnification using ImageJ software (NIH, Maryland, USA). For the mural subtype of UA cases, only areas of infiltration of tumour nests into fibrous connective tissue with at least 200 epithelial cells were analysed. Brown nuclear immunostaining was considered positive. The specimens were scored by one investigator (SP) and confirmed by an oral pathologist (CU). The Cohen’s kappa value for inter-investigator calibrations was 0.96. The positive cell staining of METTL3 was categorised as either high or low expression when the IRS scores were recorded as > 6 and ≤ 6, respectively.

-Statistical analysis

For the statistical analysis, GraphPad Prism version 10.0 (GraphPad Software, LLC., USA) was used. The mean percentage of positive cells among the odontogenic lesions was assessed using the Kruskal-Wallis test. Comparisons between the two groups were performed using Dunn’s multiple comparison test. The METTL3 percentages of positive cells, cell staining intensities, and IRS scores among the groups were compared using Yates’ chi-square test. The associations between METTL3 expression and the clinicopathological features were analysed using Fisher’s exact test. Statistical significance was defined as a *p-value* of less than 0.05.

## Results

-Demographic data and clinical characteristics of the study population

The ratio of males was higher than females in all groups. There was no statistically significant difference in gender between the groups (*p*>0.05). However, there was a statistically significant difference in the average age between the DF and DC groups, and the DF and AM groups (*p*<0.05). The mandible was the most commonly affected site, ranging from 83 to 91% depending on the lesions. Jaw swelling was observed in 30% and 100% of DC and AM cases, respectively. The majority of DC and AM cases reported no pain. Additionally, most DC cases had lesions with diameters of less than or equal to 2 cm, while most AM cases had diameters greater than 2 cm ( Table 1).

-Expression patterns of METTL3 in DF, DC, and AM

**Figure 1:**
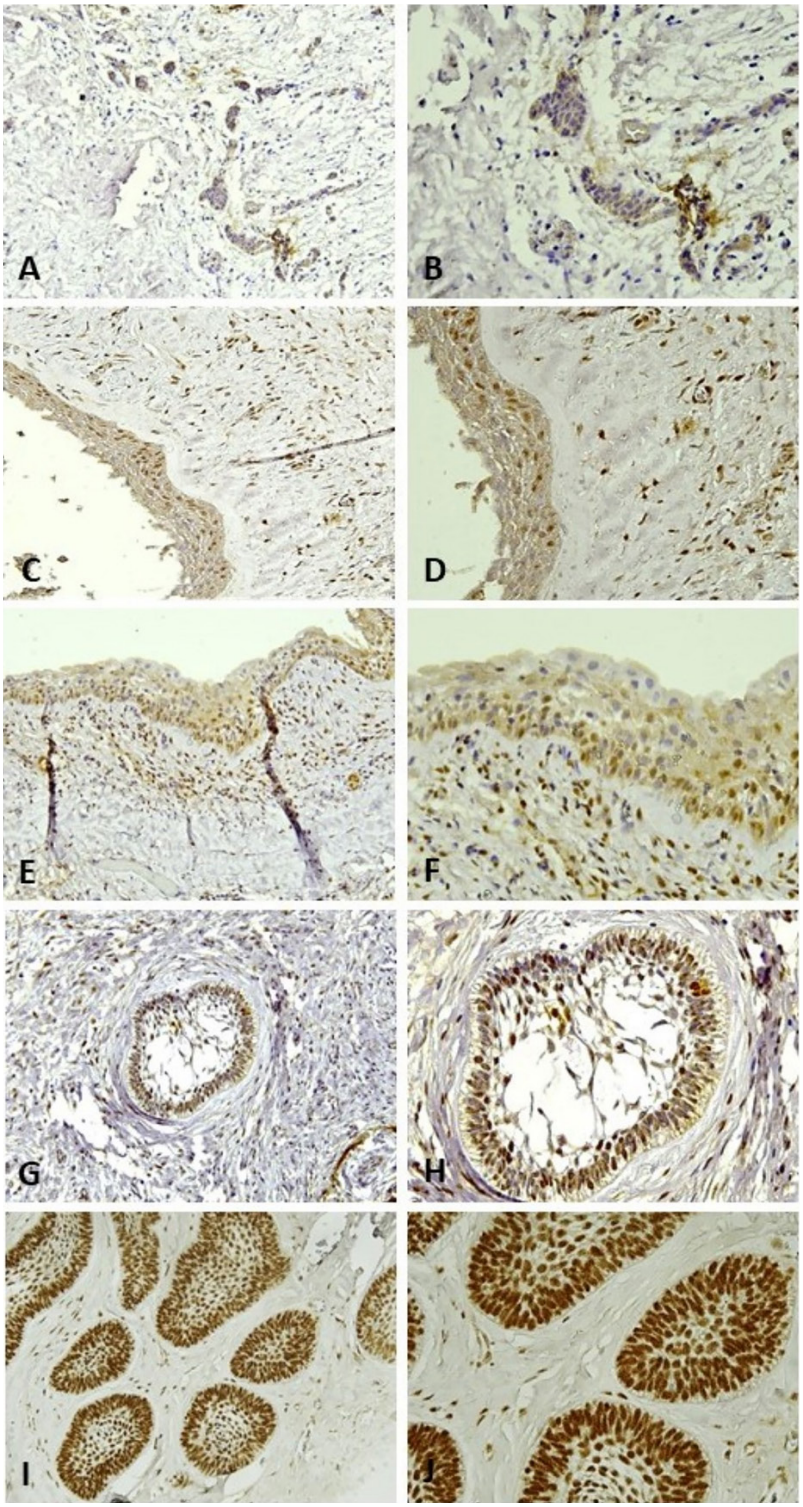
METTL3 expression in the representative cases of the DF (A, B), DC (C, D), luminal subtype of UA (E, F), mural subtype of UA (G, H), and conventional AM (I, J). In DF (A, B), no expression of METTL3 was observed in the nucleus of epithelial cells. In DC (C, D) and luminal subtype of UA (E, F), METTL3 staining was observed from the basal to upper epithelial layers and in ameloblastomatous epithelium, respectively. In mural subtype of UA (G, H) and conventional AM (I, J), METTL3 was strongly positive in the central and peripheral areas of the ameloblastic nests. Original magnification: × 200 (A, C, E, J, I), × 400 (B, D, F, H, J).

METTL3 was expressed in all samples of DC and AM, while only 42.8% of DF expressed METTL3. In the control specimen, METTL3 expression was localised in the nucleus of the human bladder tumour cells (Supplement 1) (http://www.medicinaoral.com/medoralfree01/aop/jced_62186_s01.pdf). In DF cases, METTL3 staining was negative or focally positive in the nucleus of epithelial cells with weak staining. METTL3 immunopositivity was also observed in the nucleus of epithelial cells of DC and AM. In DC cases, METTL3 expression was found at the basal, parabasal, and spinous layers of epithelium. In AM cases, METTL3 was localised in the ameloblastomatous epithelium, including in stellate-reticulum-like cells. Generally, moderate to strong METTL3 expression was observed more frequently in mural UA and conventional AM than in DC and luminal/intraluminal UA (Fig. 1A-1J).

-Percentage of METTL3-positive cells, staining intensity, and IRS score 

The mean percentage of positive METTL3 cells was the highest in AM (74.0 ± 23.5%), followed by DF (9.6 ± 13.1%) and DC (44.3 ± 16.5%). There were statistical differences in the percentages of METTL3-positive cells among the groups (*p*<0.01) with a statistical difference between the DF and DC groups (*p*<0.05), the DF and AM groups (*p*<0.01), and the DC and AM groups (*p*<0.01) ( Table 2). Among the different types of AM, the conventional AM showed the highest mean percentage of positive METTL3 cells (89.1 ± 9.5%), followed by the mural subtype of UA (68.7 ± 26.0%), and the luminal/intraluminal subtype of UA (57.6 ± 23.4%). There were statistical differences in the percentages of METTL3-positive cells among AMs (*p*<0.01) and between the luminal/intraluminal subtype of UA and conventional AM (*p*<0.01) ( Table 3).

Most AM cases (91.4%) exhibited moderate to strong METTL3 staining intensity, while most DC cases (90%) and all DF cases showed weak to moderate and negative to weak METTL3 staining intensity, respectively. There was a statistically significant difference between METTL3 staining intensity among the DF, DC, and AM groups (*p*<0.01) (Table 2) and among the AM groups (*p*<0.05) (Table 3). Most of the mural subtype of UA cases (88.9%) and all cases of conventional AM exhibited moderate to strong METTL3 staining intensity, while most of the luminal/intraluminal subtypes of UA cases (90.9%) showed weak to moderate METTL3 staining intensity (Table 3).

For the IRS score, 91.4% of AM cases exhibited moderate to strong IRS scores, while most DC cases (90%) and all DF cases showed mild to moderate and negative to mild IRS scores, respectively. There was a statistically significant difference between the IRS scores of METTL3 expression among the groups (*p*<0.01) ( Table 2). Additionally, in AMs, most of the mural subtype of UA cases (88.8%) and all cases of conventional AM exhibited moderate to strong IRS scores. Meanwhile, most of the luminal/intraluminal subtypes of UA cases (90.9%) had mild to moderate IRS scores ( Table 3). A statistical significance difference among the AM groups was also noted (*p*<0.05).

-Correlations between METTL3 immunohistochemical expression and clinical characteristics in DF, DC, and AM groups

Each group of lesions was divided into two groups, comprising 1) those with an IRS score less than or equal to 6 and 2) those with an IRS score greater than 6. The correlations between clinical characteristics and METTL3 expression in each group were analysed. There were no correlations between METTL3 expression and gender, age, location of lesions, signs of swelling, pain, and the largest diameter of lesions in orthopantomographic film in each group (*p*>0.05). However, there was a statistically significant difference in METTL3 expression between UA and conventional AM (*p*<0.05) but not in luminal and intraluminal/mural in UA and follicular/plexiform in conventional AM (*p*>0.05). These results are shown in the supplemental data in Tables 1S and 2S.

## Discussion

To the best of the authors’ knowledge, this is the first study to investigate the expression of METTL3 in odontogenic lesions. Nuclear METTL3 localisation was found in 42.8% of cases of DF and all cases of DC and AM. The pattern of METTL3 expression in the nucleus in this study was similar to what has been reported on HNSCC and OSCC (11,12,18). It was found that the percentage of METTL3-positive cells, cell staining intensity, and IRS score gradually increased from DF to DC to AM. In addition, these values were also significantly higher in the mural subtype of UA and the conventional AM compared to the luminal/intraluminal subtype of UA. The results of this study suggest that increasing METTL3 expression might be associated with a more aggressive phenotype of odontogenic lesions.

Prior studies reported that METTL3 knockdown inhibited cellular proliferation, migration, invasion, and colony formation in HNSCC, including OSCC (12,13,18,19). The knockdown of METTL3 resulted in decreasing tumour volume, weight, and lymph node metastasis rate in OSCC *in vivo* (13). According to Lin *et al*. (2022), METTL3 silencing suppressed tumour growth and decreased Ki-67 expression in pancreatic ductal adenocarcinoma in nude mice models (20). Pan *et al*. (2023) reported that overexpressing METTL3 increased the number and size of tumours as well as increased the Ki-67 cell proliferation marker in hepatocellular carcinoma *in vivo* (21). For odontogenic lesions, Ki-67 expression was shown to be greater in AM compared to DC and in conventional AM compared to UA (22). According to this study, AM had a higher METTL3 expression than DC and DF, while conventional AM had a higher METTL3 expression than UA. Taken as a whole, these data suggest that METTL3 may promote cell proliferation in odontogenic lesions through Ki-67.

Sang *et al*. (2022) reported that METTL3 knockdown could promote cell apoptosis via elevation of p21 and p53 expression in leukemic cells (23). Hypermethylation of p21 and p53 was seen in higher incidences of AM compared to DF (24). In addition, Zhang *et al*. (2021) reported that overexpression of METTL3 promoted tumour growth and the number of metastatic nodules by upregulating Bcl-2 expression in non-small cell lung cancer cells (25). It has been observed that AM expresses Bcl-2 more than DC (26), while conventional AM and the mural subtype of UA express Bcl-2 more than the luminal/intraluminal subtype of UA (27). These findings may imply that METTL3 has an anti-apoptotic role in odontogenic lesions. There has also been evidence of a genetic mutation in the MAPK/ERK pathway in AM (14). Bhattarai *et al*. (2021) reported that METTL3 overexpression increased phosphorylated RAF/MEK/ERK in melanoma cells (28). However, more research is necessary to determine the molecular mechanism of METTL3 in the pathogenesis of AM.

Increased METTL3 expression is associated with advanced T stage, poor tumour differentiation, lymphatic metastasis, and a poor overall survival rate in HNSCC and OSCC (12,13,18,19). In this study, no correlation was found between clinical characteristics and expression of METTL3 in DC, conventional AM, and UA. However, it was found that the high expression of METTL3 was correlated with conventional AM, which showed more aggressive behaviour than UA (29), suggesting the potential of METTL3 as a biomarker to predict the aggressiveness of odontogenic lesions.

This study has some limitations. First, some clinical information such as the recurrence of the lesions after treatment was missing and could not be analysed with METTL3 expression due to the retrospective nature of the study. Second, this study observed that the higher expression of METTL3 was correlated with conventional AM but not in UA, but a larger number of samples of odontogenic lesions is still required to confirm the preliminary results. Lastly, the results of this study lead to the proposition that METTL3 may be useful as a biomarker to predict the severity of odontogenic lesions, though the precise molecular mechanism of METTL3 in odontogenic cysts and tumours still needs to be elucidated.

## Conclusions

In this study, METTL3 expression was increased from DF to DC to AM. Additionally, higher expression of METTL3 was observed in conventional AM and mural subtype of UA compared to luminal/intraluminal subtype of UA. These results suggest the role of METTL3 in odontogenic lesions. Further, the expression of METTL3 may be correlated with the aggressive behaviour of these diseases.

## Figures and Tables

**Table 1:** Demographic data and clinical characteristics of the study population.

	DF^a^ (N=7)	DC^b^ (N=30)	AM^c^ (N=35)			P-value
			UA (N=20)	Conventional AM (N=15)	Total AM (N=35)	
Gender (N, %)						>0.05
Male	5 (71.4)	23 (76.7)	11 (64.7)	9 (60)	20 (57.1)	
Female	2 (28.6)	7 (23.3)	6 (35.3)	6 (40)	15 (42.9)	
Age (years old)						<0.05^abc, ab, ac^ >0.05^bc^
Range	13 – 23	15 – 77	12 – 53	13 – 68	12 – 68	
Mean + SD	18.1±3.5	39.4±18.0	30.9±11.6	39.7±18.9	34.7±15.6	
Location (N, %)						N/A
Maxilla	1 (14.2)	5 (16.7)	3 (15)	0 (0)	3 (8.6)	
Mandible	6 (85.8)	25 (83.3)	17 (85)	15 (100)	32 (91.4)	
Size in diameter^1^ (cm.) (N, %)						N/A
≤ 2	N/A	23 (76.7)	7 (35)	5 (33.3)	12 (34.3)	
> 2	N/A	7 (23.3)	13 (65)	10 (66.7)	23 (65.7)	
Swelling (N, %)						N/A
Yes	0 (0)	9 (30)	20 (100)	15 (100)	35 (100)	
No	7 (100)	21 (70)	0 (0)	0 (0)	0 (0)	
Pain (N, %)						N/A
Yes	N/A	11 (36.7)	6 (30)	2 (13.3)	8 (22.8)	
No	N/A	19 (63.3)	14 (70)	13 (86.7)	27 (77.2)	

Abbreviations: DF=dental follicle, DC= dentigerous cyst, AM=ameloblastoma, UA=unicystic ameloblastoma, N/A=not available a = DF; b = DC; c = AM; a,b,c, represent comparisons between two or more groups for statistical differences; *p*< 0.05 indicates statistically significant differences. 1 Measurement of the largest diameter of lesions in orthopantomographic film.

**Table 2:** Expression of METTL3 in dental follicle, dentigerous cyst, and ameloblastoma.

	DF^a^N = 7	DC^b^N = 30	AM^c^N = 35	P-value
Number of positive cases (N, %)	3 (42.8)	30 (100)	35 (100)	N/A
Number of negative cases (N, %)	4 (57.2)	0 (0)	0 (0)	N/A
Percentage of positive cells				
Mean ± SD	9.6 ± 13.1	44.3 ± 16.5	74.0 ± 23.5	< 0.01^abc,ac,bc^ < 0.05^ab^
Percentage of positive cells (value A) (N, %)				
Score 0 (negative)	4 (57.2)	0 (0)	0 (0)	< 0.01^abc^
Score 1 (< 10% cells)	0 (0)	0 (0)	0 (0)	
Score 2 (10 - 50% cells)	3 (42.8)	19 (63.3)	5 (14.3)	
Score 3 (> 50 - 80% cells)	0 (0)	10 (33.3)	11 (31.4)	
Score 4 (> 80% cells)	0 (0)	1 (0.4)	19 (54.3)	
Staining intensity (Value B) (N, %)				
Score 0 (negative)	4 (57.2)	0 (0)	0 (0)	< 0.01^abc^
Score 1 (Weak)	3 (42.8)	9 (30)	3 (8.6)	
Score 2 (Moderate)	0 (0)	18 (60)	17 (48.6)	
Score 3 (Strong)	0 (0)	3 (10)	15 (42.8)	
IRS score (Value A x B) (N, %)				
Score 0-1 (negative)	4 (57.2)	0 (0)	0 (0)	< 0.01^abc^
Score 2-3 (mild)	3 (42.8)	9 (30)	3 (8.6)	
Score 4-8 (moderate)	0 (0)	18 (60)	18 (51.4)	
Score 9-12 (strong)	0 (0)	3 (10)	14 (40)	

Abbreviations: DF=dental follicle, DC= dentigerous cyst, AM=ameloblastoma, N/A=not available a = DF; b = DC; c = AM; a,b,c, represent comparisons between two or more groups for statistical differences; *p*< 0.05 indicates statistically significant differences.

**Table 3:** Expression of METTL3 in different types of ameloblastoma.

	Luminal/IntraluminalUA^a^N = 11	Mural UA^b^N = 9	Conventional AM^c^N = 15	P- Value
Number of positive cases (N, %)	11 (100)	9 (100)	15 (100)	N/A
Number of negative cases (N, %)	0 (0)	0 (0)	0 (0)	N/A
Percentage of positive cells				
Mean ± SD	57.6 ± 23.4	68.7 ± 26.0	89.1 ± 9.5	< 0.01^abc, ^^ac^> 0.05^ab, ^^bc^
Percentage of positive cells (value A) (N, %)				
Score 0 (negative)	0 (0)	0 (0)	0 (0)	< 0.01^abc^
Score 1 (< 10% cells)	0 (0)	0 (0)	0 (0)	
Score 2 (10 - 50% cells)	3 (27.3)	2 (22.2)	0 (0)	
Score 3 (> 50 - 80% cells)	6 (54.5)	3 (33.3)	2 (13.3)	
Score 4 (> 80% cells)	2 (18.2)	4 (44.5)	13 (86.7)	
Staining intensity (Value B) (N, %)				
Score 0 (negative)	0 (0)	0 (0)	0 (0)	< 0.05^abc^
Score 1 (Weak)	2 (18.2)	1 (11.1)	0 (0)	
Score 2 (Moderate)	8 (72.7)	3 (33.3)	6 (40)	
Score 3 (Strong)	1 (9.1)	5 (55.6)	9 (60)	
IRS score (Value A x B) (N, %)				
Score 0-1 (negative)	0 (0)	0 (0)	0 (0)	< 0.05^abc^
Score 2-3 (mild)	2 (18.2)	1 (11.2)	0 (0)	
Score 4-8 (moderate)	8 (72.7)	4 (44.4)	6 (40)	
Score 9-12 (strong)	1 (9.1)	4 (44.4)	9 (60)	

Abbreviations: Luminal/intraluminal UA = luminal/intraluminal unicystic ameloblastoma, Mural UA= mural unicystic ameloblastoma, Conventional AM = conventional ameloblastoma; N/A=not available a = Luminal/intraluminal UA; b = Mural UA; c = Conventional AM; a,b,c represent comparisons among groups for statistical differences; *p*< 0.05 indicates statistically significant differences.

## Data Availability

The datasets used and/or analyzed during the current study are available from the corresponding author.
